# Physical Activity Behavior and Acute Myocardial Infarction, Stroke, and Sepsis Outcomes in Brazil: Insights from Targeted Eigenvector Centrality Networks

**DOI:** 10.3390/ijerph23070923

**Published:** 2026-07-18

**Authors:** Pedro Lelli Panizza, Pedro Paulo Menezes Scariot, Luana Alves Silva, Ivan Gustavo Masselli dos Reis, Leonardo Henrique Dalcheco Messias

**Affiliations:** Research Group on Technology Applied to Exercise Physiology—GTAFE, Health Sciences Postgraduate Program and Health Data Science Postgraduate Program, São Francisco University, Bragança Paulista 12916-900, SP, Brazil; drpedropanizza@gmail.com (P.L.P.); pedro.scariot@usf.edu.br (P.P.M.S.); silva.alves.luana@mail.usf.edu.br (L.A.S.); ivan.reis@usf.edu.br (I.G.M.d.R.)

**Keywords:** physical activity, complex networks, acute myocardial infarction, stroke, sepsis, eigenvector centrality, public health, Brazil

## Abstract

**Highlights:**

**Public health relevance—How does this work relate to a public health issue?**

- Acute myocardial infarction, stroke, and sepsis remain major causes of hospitalization, mortality, and healthcare expenditure in Brazil.
- Understanding how physical activity patterns relate to these conditions may support preventive strategies capable of reducing the burden on the Brazilian public health system.

**Public health significance—Why is this work of significance to public health?**

- This is the first study to simultaneously investigate acute myocardial infarction, stroke, and sepsis outcomes alongside population-level physical activity indicators using a targeted complex network approach in Brazil.
- The findings identify physical activity frequency as a more influential determinant of acute disease burden than exercise session duration.

**Public health implications—What are the key implications or messages for practitioners, policy makers and/or researchers in public health?**

- Public health initiatives should prioritize the regularity of physical activity engagement, encouraging individuals to exercise consistently throughout the week.
- Policies promoting physical activity may simultaneously contribute to reducing the burden of cardiovascular, cerebrovascular, and infectious diseases, representing a cost-effective strategy for population health improvement.

**Abstract:**

Acute myocardial infarction (AMI), stroke, and sepsis represent leading causes of hospitalization and in-hospital mortality in Brazil. Despite their substantial burden, no previous study has simultaneously examined these three conditions alongside population-level physical activity indicators in Brazil using a complex network approach. This study investigated temporal and spatial patterns in hospitalizations, hospitalization days, and deaths for AMI, stroke, and sepsis in Brazil from 2009 to 2023, integrating sociodemographic and lifestyle data from 648,460 VIGITEL survey participants. Targeted eigenvector centrality networks were constructed to identify the most systemically relevant variables associated with each outcome. For AMI and sepsis, diabetes, hypertension, and mean age consistently ranked as the most central variables, while overweight or obesity dominated the stroke networks. Physical activity emerged as a transversal component across all three conditions, with activity frequency consistently outranking session duration in all nine network analyses performed. These findings suggest that the regularity of physical activity engagement carries greater population-level protective relevance than session length, highlighting physical activity promotion as a cost-effective public health strategy for simultaneously reducing the burden of cardiovascular, cerebrovascular, and infectious acute disease in Brazil.

## 1. Introduction

Acute myocardial infarction (AMI), stroke, and sepsis represent three of the most burdensome acute clinical conditions in terms of hospitalizations, in-hospital mortality, and healthcare resource utilization worldwide [1,2,3]. Despite their distinct pathophysiological mechanisms, these conditions share common determinants rooted in chronic low-grade inflammation, endothelial dysfunction, and dysregulation of cardiovascular and immune homeostasis [4,5,6,7]. The global epidemiological transition toward an aging population with increasing prevalence of sedentary behavior, obesity, and metabolic syndrome has progressively amplified the incidence and severity of these conditions, imposing a disproportionate burden on healthcare systems, especially in low- and middle-income countries [8,9,10]. Physical inactivity, now recognized as a major independent risk factor for cardiovascular and infectious disease outcomes [11,12,13], contributes to the systemic vulnerability that underlies both the onset and the severity of AMI, stroke, and sepsis, making the population-level distribution of physical activity a critical determinant of acute hospital burden.

Brazil’s continental dimensions and the structural heterogeneity of its Unified Health System (SUS) generate a complex epidemiological landscape in which outcomes for time-sensitive acute conditions are highly sensitive to regional infrastructure, workforce availability, and proximity to tertiary care [14,15]. The five macroregions of the country differ substantially in demographic profiles, prevalence of cardiovascular risk factors, habitual physical activity levels, and hospital capacity [16,17,18]. In-hospital mortality from AMI and stroke has been consistently shown to vary by geographic region [19,20], while sepsis-related outcomes are critically dependent on the availability of intensive care unit beds and early clinical recognition protocols [21]. Importantly, regional differences in physical activity and sedentary behavior partially mirror these outcome disparities [22,23], suggesting that the population-level physiological profile of each macroregion may be reflected in the epidemiological indicators of acute disease. Longitudinal surveillance of hospitalization rates, days, and deaths across such heterogeneous territories is therefore essential not only for epidemiological monitoring but also for informing targeted public health interventions aimed at disease prevention, health promotion, and the encouragement of active lifestyles.

Although traditional epidemiological analyses have provided substantial insight into the burden of acute conditions in Brazil, these approaches typically treat each disease in isolation and rely on linear statistical frameworks. Complex network models offer a compelling alternative, as they allow the representation of multiple variables as interconnected nodes whose relational structure can reveal emergent patterns not discernible through conventional analyses [24,25,26]. When applied to health data aggregated by geographic region and time, network-based approaches may enable the identification of clusters, hubs, and dynamic transitions in population health indicators, providing a richer and more integrative picture of how acute disease outcomes and their physiological determinants co-evolve across space and time. Importantly, complex network models are constructed from the relational structure among variables rather than from spatial contrasts between units, meaning that regional data need not be analyzed in isolation but can instead be pooled to maximize the number of observations available for edge estimation.

In this context, the present study aimed to investigate temporal and spatial patterns in hospitalizations, hospitalization days, and deaths related to AMI, stroke, and sepsis across the five Brazilian macroregions between 2009 and 2023, together with population-level physical activity indicators. The simultaneous evaluation of these three acute conditions through a longitudinal complex network approach addresses an important gap in the Brazilian epidemiological literature. Although the beneficial role of physical activity has been widely investigated for several chronic health outcomes, its structural relationships with different acute disease burdens at the population level remain poorly understood. Therefore, we hypothesized that physical activity indicators would occupy central positions within the resulting network structures and that the three acute conditions would exhibit distinct topological organizations, reflecting differences in their epidemiological profiles across Brazil.

## 2. Materials and Methods

### 2.1. Study Design

A longitudinal ecological study was conducted using aggregated population-level data spanning fifteen years, from 2009 to 2023. Although data were originally organized according to the five Brazilian macroregions (North, Northeast, Central-West, Southeast, and South), all variables were subsequently pooled at the national level to constitute the analytical dataset. This aggregation strategy was adopted to maximize the number of observations available for network estimation, thereby increasing the statistical robustness of the relational structure modeled. Reporting followed the RECORD [27] guidelines for observational studies using routinely collected health data, and all data accessed were fully anonymized and freely available in public repositories, requiring no ethical approval for secondary data use under current Brazilian legislation.

### 2.2. Data Source

The study drew on two complementary national surveillance infrastructures. Information on acute inpatient events was sourced from the Hospital Information System (SIH/SUS), a nationwide administrative database maintained by the Brazilian Ministry of Health that captures all hospitalizations reimbursed by the public health system. Behavioral and lifestyle exposure data were obtained from VIGITEL (Surveillance System for Risk and Protective Factors for Chronic Diseases by Telephone Survey), an annual telephone-based survey conducted across all Brazilian state capitals and the Federal District that tracks the prevalence of health-related behaviors in the adult population. The physical activity module of VIGITEL is based on standardized questionnaires developed by the Brazilian Ministry of Health and has demonstrated acceptable validity and reproducibility for population-based surveillance of leisure-time physical activity among Brazilian adults [28,29].

### 2.3. Variables, Data Handling, and Analytical Considerations

Four outcome indicators were extracted from SIH/SUS for each of the three clinical conditions under investigation, that is, AMI, stroke, and sepsis. For each condition, the annual count of hospitalizations, hospitalization days, and the total number of in-hospital deaths were retrieved. Behavioral and clinical indicators were derived from VIGITEL and comprised nine variables, including mean age of the surveyed population (expressed as a continuous value in years) and eight prevalence estimates (%), namely, the proportion of individuals with higher education (university level), prevalence of overweight or obesity, prevalence of physical activity, proportion of adults engaging in physical activity for 30 min or more per session, proportion engaging in physical activity on three to four days per week, smoking prevalence, prevalence of self-reported hypertension, and prevalence of self-reported diabetes. Each estimate was recorded per macroregion per year, such that every region–year combination constituted an independent observational unit (N = 1), yielding a dataset in which the five macroregions observed across fifteen consecutive years generated the full sample used for network modeling (N = 75). All variables were subsequently pooled at the national level and treated as nodes within the complex network structure.

### 2.4. Complex Networks and Statistical Methods

Inspired by previous studies [30,31,32,33,34,35], weighted and targeted complex networks were constructed to identify variables with the greatest systemic influence on each outcome of interest (i.e., AMI, stroke, and sepsis hospitalizations, hospitalization days, and deaths). The analytical framework was based on targeted eigenvector centrality, in which the centrality of each node reflects both the strength of its direct connection to the target outcome and the centrality of its neighboring nodes within the network topology.

Prior to network construction, Spearman’s rank correlation coefficients were computed between all variable pairs, given the non-normal distribution of the data confirmed by the Shapiro–Wilk test. To reduce the likelihood of false-positive edges resulting from multiple pairwise comparisons, *p*-values were adjusted using the Benjamini–Hochberg false discovery rate (FDR) procedure, and only correlations remaining statistically significant after correction were retained as network edges. After applying this correction, 249 of the 267 edges originally retained across the nine main networks remained statistically significant. The excluded edges were weak indirect associations and did not alter the main target-outcome connections. Centrality rankings were highly stable after correction, with median Kendall’s τ = 1.00 and median Spearman’s ρ = 1.00 between the original and FDR-adjusted rankings. Thus, the main network topology and the interpretation of the principal findings were preserved. Edge weights were determined by the product of two factors: Spearman’s correlation coefficient and a geometric proximity weighting factor. The proximity degree to the target node was assigned as 1.0 for direct connections, 0.5 for second-degree, 0.25 for third-degree, 0.125 for fourth-degree, and 0.0625 for fifth-degree connections. This geometric decay scheme was adopted to progressively reduce the contribution of increasingly indirect connections while preserving the greater influence of variables located closer to the target outcome.

The eigenvector centrality scores were computed using Python (version 3.9.3) with the NetworkX 2.5 library [36] (Supplementary File S1). Results are expressed as targeted eigenvector centrality values ranging from 0 to 1, with higher scores indicating greater systemic relevance to the target outcome. Graphical representations were generated using ggplot2 from the R statistical software version 4.5.2 (R Foundation for Statistical Computing, Vienna, Austria).

## 3. Results

**Figure 1:**
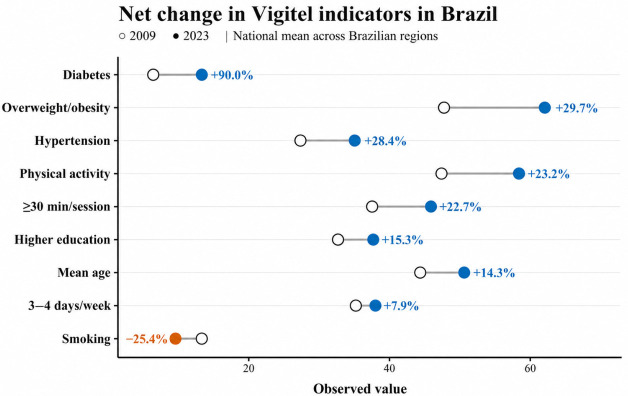
Demographic, behavioral, and cardiometabolic indicators obtained from the Brazilian Surveillance System of Risk and Protective Factors for Chronic Diseases by Telephone Survey (VIGITEL) between 2009 and 2023. Open circles represent 2009, and filled circles represent 2023. Lines indicate changes over time, and percentages represent the relative change between 2009 and 2023. Mean age is expressed in years; all other variables are presented as prevalence (%). Open circles represent values observed in 2009, filled circles represent values observed in 2023, blue indicates an increase, and orange indicates a decrease.

Sociodemographic and lifestyle variables from VIGITEL comprised 648,460 individuals interviewed across 32 Brazilian state capitals over the same 15-year period. The prevalence of overweight or obesity was the most prominent behavioral risk factor across all regions, with values consistently exceeding 50% in most region–year strata. Current smoking prevalence was relatively low, ranging from approximately 5% to 17% across regions. Hypertension affected roughly 28–47% of the surveyed population, while diabetes prevalence ranged from 6% to 20%. Physical activity participation was reported by approximately 45–60% of respondents, with 30–47% engaging in sessions of at least 30 min and 32–42% exercising three to four days per week. College-level education was attained by approximately 28–44% of the sample across regions (Figure 1).

**Figure 2:**
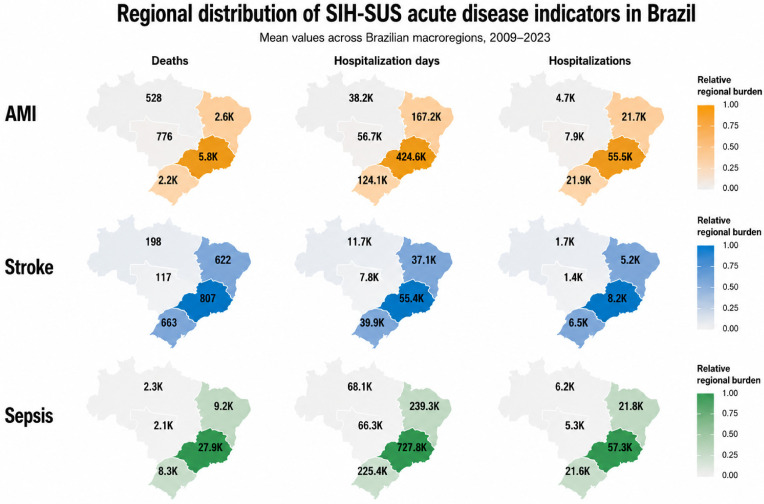
Regional distribution of acute myocardial infarction (AMI), stroke, and sepsis burden in Brazil according to the Hospital Information System of the Brazilian Unified Health System (SIH-SUS) between 2009 and 2023.

**Figure 3:**
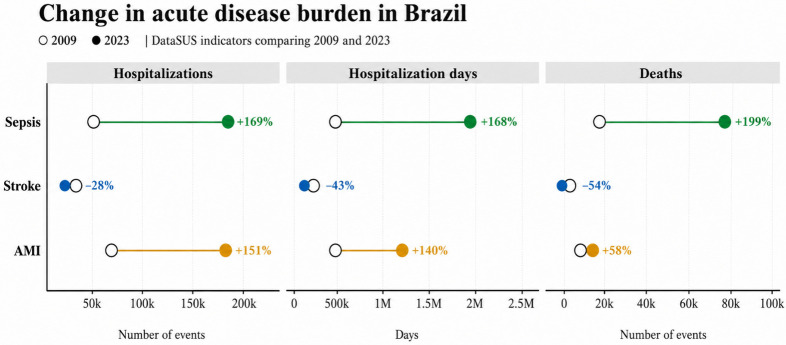
Hospitalizations, hospitalization days, and deaths associated with acute myocardial infarction (AMI), stroke, and sepsis comparing 2009 and 2023 in Brazil. DataSUS/SIH-SUS: Hospital Information System of the Brazilian Unified Health System. Green indicates sepsis, blue indicates stroke, and orange indicates AMI. Open circles represent 2009, filled circles represent 2023, and the connecting lines indicate the change between the two years.

Regarding SIH/SUS, AMI accounted for 2,110,194 hospitalizations, 14,980,708 hospitalization days, and 213,989 deaths nationwide, while stroke resulted in 417,285 hospitalizations, 2,752,912 hospitalization days, and 43,711 deaths. Not less importantly, sepsis registered 2,102,323 hospitalizations, 24,676,734 hospitalization days, and 930,686 in-hospital deaths, representing the greatest overall hospital burden among the three acute conditions evaluated. A complete regional summary of hospitalizations, hospitalization days, and deaths for the three conditions is provided in Table 1, whereas their geographic distribution is illustrated in Figure 2. The temporal changes in acute disease burden in Brazil between 2009 and 2023 are presented in Figure 3.

**Figure 4:**
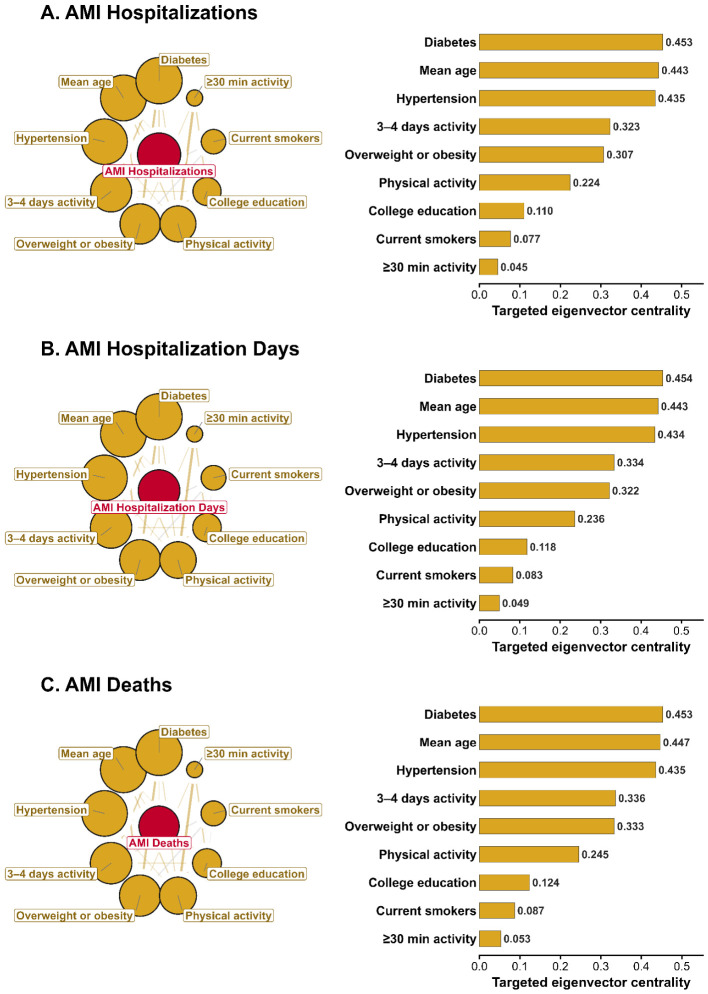
Targeted eigenvector networks for acute myocardial infarction (AMI). Circular networks (**left**) and targeted eigenvector centrality rankings (**right**) of sociodemographic and lifestyle factors associated with AMI hospitalizations (**A**), hospitalization days (**B**), and deaths (**C**) in Brazil.

For the AMI network (Figure 4), diabetes consistently ranked as the variable with the highest targeted eigenvector centrality across all three panels. Mean age ranked second across all three panels (0.443, 0.443, and 0.447, respectively), followed closely by hypertension (0.435, 0.434, and 0.435). Overweight or obesity ranked fifth for hospitalizations (0.307) and hospitalization days (0.322), rising slightly to fourth for deaths (0.333). Physical activity ranked sixth across all three AMI panels (0.224, 0.236, and 0.245), occupying a consistent mid-tier position and outranking college education, current smokers, and ≥30 min activity in all outcomes. The ≥30 min activity variable ranked last across all AMI panels (0.045, 0.049, and 0.053), and 3–4 days of activity ranked fourth for hospitalizations and hospitalization days (0.323 and 0.334) and third for deaths (0.336).

**Figure 5:**
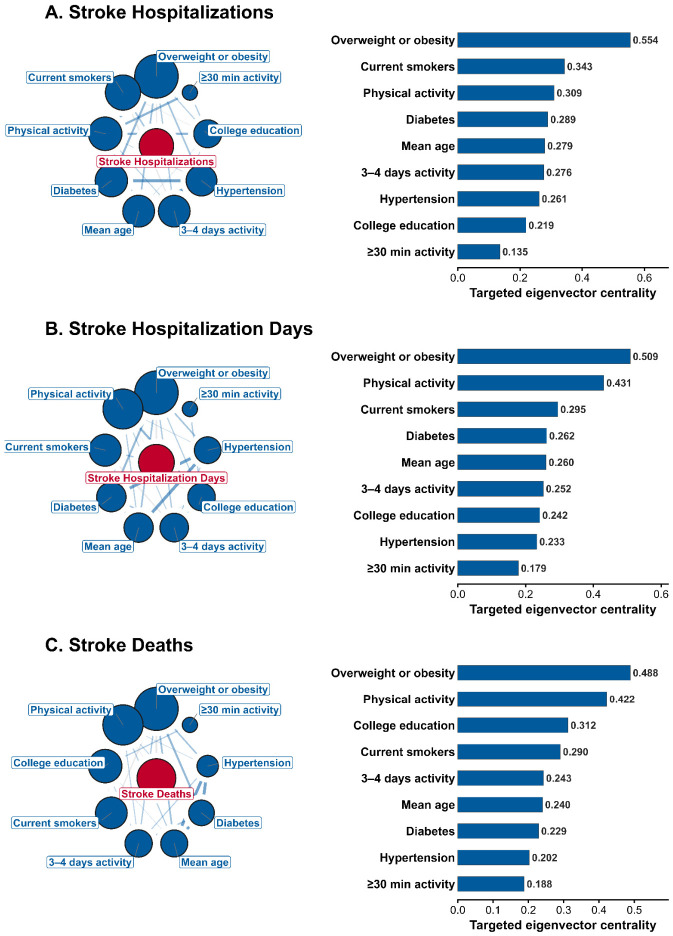
Targeted eigenvector networks for stroke. Circular networks (**left**) and targeted eigenvector centrality rankings (**right**) of sociodemographic and lifestyle factors associated with stroke hospitalizations (**A**), hospitalization days (**B**), and deaths (**C**) in Brazil.

For stroke outcomes (Figure 5), overweight or obesity emerged as the most systemically relevant variable across all three panels. Current smokers ranked second for hospitalizations (0.343), while physical activity assumed that position for hospitalization days (0.431) and deaths (0.422). College education showed an increase in centrality for stroke deaths (0.312), becoming the third most central variable in that panel. Hypertension ranked seventh for hospitalizations (0.261) but dropped to eighth for deaths (0.202). Of particular relevance, physical activity demonstrated the strongest prominence among all physical activity-related variables across stroke outcomes, ranking second for both hospitalization days and deaths, with 3–4 days of activity ranking sixth across all panels (0.276, 0.252, and 0.243) and ≥30 min activity consistently occupying the last position (0.135, 0.179, and 0.188).

**Figure 6:**
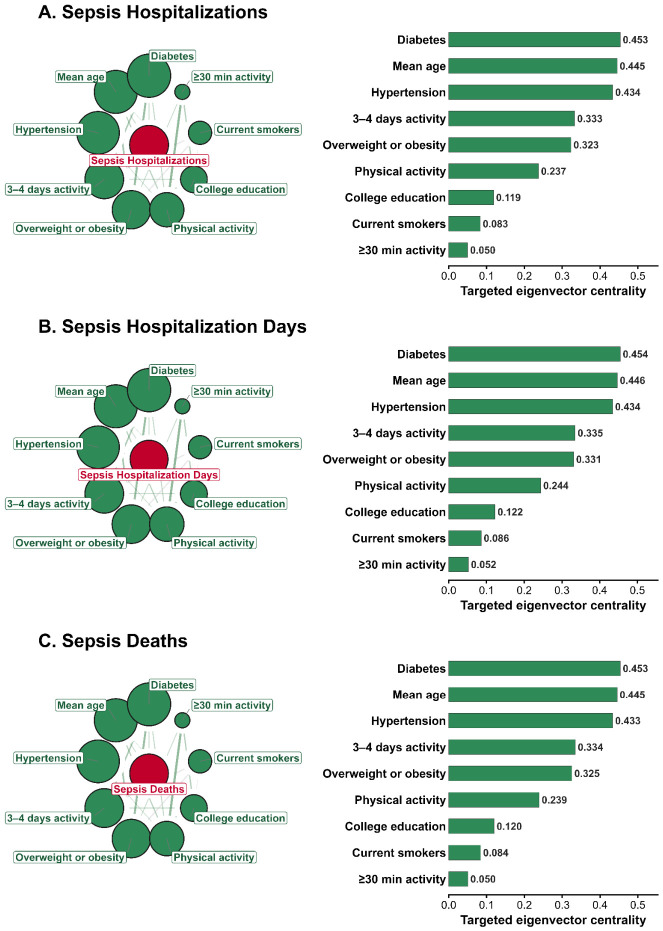
Targeted eigenvector networks for sepsis. Circular networks (**left**) and targeted eigenvector centrality rankings (**right**) of sociodemographic and lifestyle factors associated with sepsis hospitalizations (**A**), hospitalization days (**B**), and deaths (**C**) in Brazil.

Regarding sepsis outcomes (Figure 6), the network structure closely mirrored that observed for AMI, with diabetes ranking first across all three panels, followed by mean age and hypertension. Overweight or obesity ranked fifth across all sepsis panels (0.323, 0.331, and 0.325). For physical activity indicators, physical activity ranked sixth across all three sepsis panels (0.237, 0.244, and 0.239), while 3–4 days of activity ranked fourth (0.333, 0.335, and 0.334), consistently outranking overweight or obesity in the hospitalization and hospitalization day panels. The ≥30 min activity variable again presented the lowest centrality scores across all sepsis outcomes (0.050, 0.052, and 0.050).

## 4. Discussion

The present study employed targeted eigenvector centrality networks to investigate the structural associations between sociodemographic, behavioral, and clinical variables and the burden of AMI, stroke, and sepsis across Brazil between 2009 and 2023. We hypothesized that physical activity indicators would occupy central positions within the resulting network structures and that distinct topological patterns would characterize each acute condition. Overall, our findings supported this hypothesis. Physical activity consistently occupied central positions across all nine network analyses, with participation frequency emerging as a more central dimension than session duration. Furthermore, distinct condition-specific topological profiles were identified, with diabetes, hypertension, and mean age predominating in the AMI and sepsis networks, whereas overweight or obesity occupied the highest-ranking positions in the stroke networks.

The central role of physical activity in the present findings warrants careful interpretation in the context of Brazilian public health. Physical activity ranked second for both stroke hospitalization days and stroke deaths, positioning it alongside overweight or obesity as a primary systemic determinant of stroke burden at the population level. For AMI and sepsis, physical activity ranked sixth across all panels, reflecting a secondary but consistent systemic influence. More strikingly, the 3–4 days of activity variable consistently outranked general physical activity participation in both AMI and sepsis networks. In AMI, it ranked fourth for hospitalizations and hospitalization days and third for deaths, whereas in sepsis, it consistently occupied the fourth position across all three outcome panels. In contrast, the ≥30 min activity variable systematically occupied the last position across all nine analyses. This dissociation between frequency and duration is a key finding of the present study and carries direct implications for how physical activity guidelines are framed and communicated at the population level. Current recommendations typically emphasize achieving a minimum of 150 min of moderate-intensity physical activity per week, often operationalized as sessions of at least 30 min [12,37]. The present findings, however, suggest that the regularity of engagement was more associated with network centrality than the duration of individual sessions, at least within the targeted network framework adopted in this study. This interpretation aligns with emerging evidence suggesting that habitual physical activity, even at moderate durations, produces sustained adaptations in cardiovascular autonomic regulation, systemic inflammation, endothelial function, and immune competence that are not fully replicated by infrequent but prolonged effort bouts [13,38,39,40]. In this sense, the consistency of physical activity over time appeared to be more associated with the overall network structure than indicators reflecting the intensity or duration of individual sessions, a pattern that may warrant greater attention in population-level physical activity surveillance and future epidemiological studies.

The biological mechanisms underpinning the systemic relevance of physical activity observed across all three conditions are multifaceted. For stroke, regular physical activity is associated with neuroprotective and cerebrovascular effects through reductions in blood pressure, attenuation of arterial stiffness, improvements in cerebral perfusion, and modulation of inflammatory and thrombotic pathways [11,41,42,43]. Further, these factors are mechanistically linked to the leading risk factors identified in the stroke network, including overweight or obesity and current smoking [44,45,46]. The prominence of physical activity in the panels related to stroke hospitalization days and deaths, rather than solely in hospitalizations, further suggests that habitual physical activity behavior was more associated with indicators reflecting stroke burden than with hospitalization frequency alone. This pattern is consistent with the hypothesis that pre-stroke cardiorespiratory fitness may be related to more favorable clinical trajectories, although such mechanisms cannot be inferred from the present ecological analysis. For AMI, the sixth-rank position of physical activity across all panels, combined with the fourth-rank position of 3–4 days of activity for deaths, may reflect a well-established protective role of regular physical activity against acute coronary events, mediated through improvements in lipid profiles, insulin sensitivity, myocardial perfusion reserve, and autonomic nervous system balance [47,48]. The finding that physical activity frequency ranked above overall participation in the AMI death network suggests that regular engagement in physical activity occupied a central position within the network, highlighting its association with the observed epidemiological structure.

For sepsis, the systemic relevance of physical activity is perhaps the most clinically underrecognized finding of the present study. The consistent sixth-rank position of physical activity and fourth-rank position of the 3–4 days/week indicator across the sepsis panels may indicate that regular engagement was consistently associated with population-level sepsis burden. This pattern is consistent with mechanisms proposed in previous studies involving immune surveillance, anti-inflammatory cytokine modulation, and mitochondrial function, although such mechanisms cannot be inferred from the present ecological analysis [49,50,51,52]. This finding adds to a growing body of literature supporting the immunomodulatory role of regular physical activity and highlights sepsis as an underexplored outcome in this context. Importantly, the striking similarity between the AMI and sepsis network topologies is not coincidental and can be explained by the near-perfect correlation between the outcome indicators of these two conditions across the dataset. Spearman correlation analyses revealed coefficients between AMI and sepsis for hospitalizations (r = 0.97; *p* < 0.001), hospitalization days (r = 0.98; *p* < 0.001), and deaths (r = 0.95; *p* < 0.001). This co-occurrence likely reflects shared upstream determinants that simultaneously predispose populations to acute coronary events and to the immune dysregulation underlying sepsis susceptibility, suggesting the notion that both conditions are, to a considerable extent, expressions of the same chronic disease burden at the population level [53,54].

The condition-specific findings beyond physical activity also deserve discussion. The dominance of diabetes, hypertension, and mean age in the AMI and sepsis networks may reflect a shared systemic vulnerability profile associated with chronic cardiometabolic conditions and biological aging. Diabetes contributes to endothelial dysfunction, accelerated atherosclerosis, and impaired immune clearance, establishing direct mechanistic pathways to both acute coronary events and sepsis susceptibility [55,56,57]. Hypertension promotes structural cardiac remodeling and heightens myocardial oxygen demand, while simultaneously impairing microvascular perfusion relevant to infection control [58,59]. The convergence of these two chronic conditions (i.e., diabetes and hypertension) at the top of both the AMI and sepsis centrality rankings may indicate that integrated cardiometabolic risk management deserves particular attention in future epidemiological studies and public health planning. For stroke, the emergence of overweight or obesity as the leading variable across all three outcome panels likely reflects its role as a network hub, simultaneously connected to hypertension, diabetes, physical inactivity, and dyslipidemia within the broader risk factor topology, thereby amplifying its eigenvector centrality beyond what direct associations alone would suggest. The rise of college education to the third position in the stroke deaths panel further reinforces the relevance of socioeconomic determinants in stroke outcomes [60,61,62], consistent with evidence linking lower educational attainment to delayed recognition of stroke symptoms, reduced access to acute reperfusion therapies, and higher post-stroke mortality.

The national epidemiological panorama described in this study contextualizes these network findings within a broader public health scenario. The progressive increase in AMI hospitalizations across all macroregions, contrasting with declining stroke hospitalizations, may suggest improvements in acute stroke management and prevention while indicating that AMI primary prevention remains insufficient in Brazil. The regional disparities observed throughout the study period may reflect structural inequalities in healthcare access and infrastructure that persist despite national health system expansion.

These regional differences should also be interpreted within the broader socioeconomic and demographic heterogeneity of Brazil. The Southeast and South regions concentrate a larger proportion of the country’s older population, greater urbanization, and higher availability of specialized healthcare services, factors that may increase both the detection and treatment of acute conditions while simultaneously reflecting a greater burden of chronic cardiometabolic diseases. In contrast, the North and parts of the Northeast face persistent challenges related to healthcare accessibility, geographic barriers, and socioeconomic inequalities, which may contribute to differences in disease management and hospitalization patterns. Consequently, the regional network structures identified in the present study likely reflect not only differences in behavioral risk factors but also broader contextual determinants operating at the population level. Within this context, the consistent systemic relevance of physical activity indicators may suggest the potential of population-level physical activity promotion as a transversal public health strategy capable of simultaneously attenuating the burden of cardiovascular, cerebrovascular, and acute infectious diseases. Given that Brazil faces the double burden of increasing chronic disease prevalence alongside persistently high acute care demand [63], investments in policies that promote regular physical activity engagement represent a cost-effective and evidence-based priority for the national public health agenda.

The present study has limitations that warrant consideration. The ecological design, with data aggregated at the macroregional level, precludes individual-level causal inference and may obscure within-region heterogeneity in both risk factor prevalence and disease outcomes. In addition, although each region–year combination was treated as an observational unit for network construction, consecutive observations within the same macroregion are likely to exhibit temporal dependence because demographic, behavioral, and health indicators evolve progressively over time. Consequently, the effective statistical independence of the analytical dataset may be lower than the nominal number of region–year observations, which should be considered when interpreting the estimated correlation structure. Nevertheless, the objective of the present study was to characterize the overall relational structure among variables rather than to perform formal longitudinal inference, and future studies should explore network approaches capable of explicitly accounting for within-region temporal correlation. The VIGITEL survey does not capture populations residing in rural or interior municipalities, potentially underestimating lifestyle-related risk factor burden in less urbanized areas. Furthermore, the network centrality approach is sensitive to the selection of variables included in the analysis and does not account for unmeasured confounders such as dietary patterns, socioeconomic deprivation, or healthcare access.

An additional limitation should be considered when interpreting the present findings. To enable the integration of SIH/SUS hospital indicators with VIGITEL population-based data, analyses were performed using region-level aggregated and prevalence-based estimates rather than absolute population counts. Consequently, direct comparisons with studies based on individual-level data, incidence rates, or standardized epidemiological indicators should be interpreted with caution. The network structures generated in the present study reflect relative associations among variables within the integrated dataset rather than direct estimates of disease risk or causal effects. Nevertheless, this approach was necessary to allow the simultaneous exploration of hospital burden and population-level behavioral characteristics within a unified analytical framework. Equally important, the strong correlation between AMI and sepsis outcomes should be acknowledged when interpreting the network analyses. Although separate targeted networks were constructed for each clinical condition to preserve disease-specific interpretation, the high correlation between these hospitalization indicators resulted in highly similar network topologies. Consequently, the AMI and sepsis networks should be interpreted as complementary rather than entirely independent representations of the underlying epidemiological structure. Future studies incorporating a broader range of acute conditions and multivariate network approaches may help further disentangle disease-specific from shared systemic network patterns.

Conversely, the simultaneous analysis of three acute conditions across 15 years and five macroregions using a complex network framework represents a methodological strength, enabling the identification of transversal and condition-specific patterns that would not emerge from conventional regression-based approaches. The use of targeted eigenvector centrality, rather than generic network metrics, allowed for outcome-specific interpretations of variable relevance, increasing the clinical and epidemiological applicability of the findings. The geometric decay weighting adopted in the targeted eigenvector analysis reflects a predefined methodological assumption regarding the progressively decreasing contribution of indirect connections. Although this weighting strategy has been consistently employed in our previous studies, future methodological investigations should formally compare alternative decay functions to evaluate their impact on network topology and node rankings. Further studies should also explore individual-level data linkages between VIGITEL, SIH/SUS, and other national health registries to enable longitudinal and multilevel analyses and should expand the variable set to include nutritional, genetic, and healthcare access indicators, as well as objective measures of physical activity, to further elucidate the network determinants of acute disease burden in Brazil.

## 5. Conclusions

The present study demonstrated that physical activity, particularly its frequency dimension, was consistently associated with the network structure of AMI, stroke, and sepsis across Brazil, as revealed by targeted eigenvector centrality network analysis. Furthermore, the network topologies indicated that indicators reflecting the regularity of physical activity engagement consistently occupied more central positions than indicators describing the duration of individual sessions. Beyond physical activity, distinct epidemiological profiles were observed for each condition, with diabetes, hypertension, and mean age emerging as the most central variables in the AMI and sepsis networks, whereas overweight and obesity occupied the most central positions within the stroke networks. Collectively, these findings support the value of complex network modeling as a complementary epidemiological tool for exploring relationships among population-level health indicators and suggest that regular physical activity deserves further attention in future epidemiological research and public health planning aimed at addressing the burden of acute cardiovascular, cerebrovascular, and infectious diseases in Brazil.

## Figures and Tables

**Table 1:** Cumulative hospital burden of acute myocardial infarction (AMI), stroke, and sepsis across the five Brazilian macroregions according to SIH/SUS (2009–2023).

Region	Hospitalizations (n)	Hospitalization Days (Days)	Deaths (n)
**AMI**			
North	89,868	729,287	9459
Northeast	409,650	3,108,231	46,030
Southeast	1,046,887	7,769,131	104,980
South	409,427	2,315,713	39,366
Central-West	154,362	1,058,346	14,154
Brazil	2,110,194	14,980,708	213,989
**Stroke**			
North	31,286	218,772	3761
Northeast	92,223	657,485	11,357
Southeast	151,983	1,019,213	14,670
South	115,740	713,107	11,750
Central-West	26,053	144,335	2,173
Brazil	417,285	2,752,912	43,711
**Sepsis**			
North	115,641	1,249,314	42,299
Northeast	401,762	4,439,843	170,470
Southeast	1,071,047	13,461,178	518,927
South	410,159	4,246,664	157,359
Central-West	103,714	1,279,735	41,631
Brazil	2,102,323	24,676,734	930,686

## Data Availability

The original contributions presented in the study are included in the article. All data can be obtained from VIGITEL and SIH/SUS.

## Supplementary Materials

The following supporting information can be downloaded at: https://www.mdpi.com/article/10.3390/ijerph23070923/s1, File S1: Imported python libraries.
