# Supplementary material for: Physical Activity Behavior and Acute Myocardial Infarction, Stroke, and Sepsis Outcomes in Brazil: Insights from Targeted Eigenvector Centrality Networks
Source: Int J Environ Res Public Health. 2026 Jul 18;23(7):923. doi: 10.3390/ijerph23070923 (PMC13410189; doi:10.3390/ijerph23070923)
Supplement: Supplementary file 1 [file ijerph-23-00923-s001.zip › ijerph-4396378-supplementary.pdf]

File S1. IMPORTED PYTHON LIBRARIES

```
import pandas as pd

from scipy.stats import pearsonr

import numpy as np

from IPython.display import clear_output

import networkx as nx
```

FUNCTION USED TO CALCULTE PEARSON CORRELATIONS

```
def calculateCor(df):

    rho = df.corr()

    pval = df.corr(method=lambda x, y: pearsonr(x, y)[1]) - np.eye(*rho.shape)

    p = pval.map(lambda x: ''.join(['*' for t in [0.001, 0.01, 0.05] if x <= t]))

    return pval, rho.round(2).astype(str) + p
```

FUNCTION USED TO GET A DATA FRAME WITH THE PAIRWISES OF VARIABLE LABELS THAT ARE SIGNIFICANTLY CORRELATED

```
def organizeCorr(df):

    df_corr = pd.DataFrame()

    for i, col in enumerate(df):

        temp = df[col]

        #temp.drop(labels=col, axis=0, inplace=True)

        list_destiny = []

        for j, row in enumerate(temp):
```

```
        if (row < 0.05) and (row > -0.05) and col != df.index[j]:
            list_destiny.append(df.index[j])
    df_corr = pd.concat([df_corr, pd.DataFrame({df.index[i]: list_destiny})], axis=1)
  return df_corr.dropna(axis=1, how='all')
```

FUNCTION USED TO GET A DATA FRAME WITH THE CORRELATION COEFICIENTS BETWEEN VARIABLES THAT ARE SIGNIFICANTLY CORRELATED

```
def organizeCoef(df1, df2):
  df_coef = pd.DataFrame()
  for i, col in enumerate(df1):
    temp = df1[col]
    list_destiny = []
    for j, row in enumerate(temp):
        if (row < 0.05) and (row > -0.05) and col != df1.index[j]:
            list_destiny.append(df2.iloc[i, j])
    df_coef = pd.concat([df_coef, pd.DataFrame({df1.index[i]: list_destiny})], axis=1)
  return df_coef.dropna(axis=1, how='all')
```

FUNCTION USED TO BUILD A DATA FRAME WITH THE INPUT FORMAT TO BE CALLED IN THE WEIGHTS CALCULATION

```
def formatCoef(df_corr, df_coef, target):
  col_coef = []
  col_sig = []
```

```
    temp = df_corr[target].dropna()
    for i in range(len(temp)):
        txt = df_coef[target][i]
        coef = float(txt.split('*', 2)[0])
        col_coef.append(abs(coef))
        if (coef < 0):
            col_sig.append('-')
        else:
            col_sig.append('+')
    return pd.DataFrame({"coef": col_coef}), pd.DataFrame({"signal": col_sig})
```

FUNCTION USED TO CALCULATION OF WEIGHTS

```
def calculateWeights(df_corr, df_coef, target):
    weight = 1
    col_source = pd.DataFrame({"source": range(len(df_corr[target].dropna()))}).astype(str)
    col_source[col_source.astype(int) >= 0] = target
    col_destiny = pd.DataFrame({"destiny": df_corr[target].dropna()})
    col_weight = pd.DataFrame({"weight": range(len(df_corr[target].dropna()))})
    col_weight[col_weight >= 0] = 1
    col_coef, col_sig = formatCoef(df_corr, df_coef, target)
    counter1 = 0
    size = df_corr.shape[1]
    while(True):
```

```
counter1 += 1
counter2 = 0
temp1 = []
temp2 = []
temp3 = []
temp4 = []
temp5 = []
weight = weight / 2
last_col = col_destiny.iloc[:, -1:].dropna()
steps = len(last_col)
for i, row1 in enumerate(last_col.iloc[:, 0]):
    counter2 += 1
    if row1 not in col_source.values and row1 not in temp1 and row1 != temp1[-1:]:
        for j, row2 in enumerate(df_corr[row1].dropna()):
            if row2 not in col_source.values and row2 not in temp1:
                temp1.append(row1)
                temp2.append(row2)
                temp3.append(weight)
                txt = df_coef[row1].dropna()[j]
                coef = float(txt.split('*', 2)[0])
                temp4.append(abs(coef))
                if (coef < 0):
                    temp5.append('-')
```

```
            else:
                temp5.append('+')
        clear_output()
        print("step", counter1, "of", size, ":", round(counter2/steps * 100, 2), "% done")
    if (temp1):
        col_source = pd.concat([col_source, pd.DataFrame({"source": temp1})], axis=0).reset_index(drop=True)
        col_destiny = pd.concat([col_destiny, pd.DataFrame({"destiny": temp2})], axis=0).reset_index(drop=True)
        col_weight = pd.concat([col_weight, pd.DataFrame({"weight": temp3})], axis=0).reset_index(drop=True)
        col_coef = pd.concat([col_coef, pd.DataFrame({"coef": temp4})], axis=0).reset_index(drop=True)
        col_sig = pd.concat([col_sig, pd.DataFrame({"signal": temp5})], axis=0).reset_index(drop=True)
    else:
        return col_source, col_destiny, col_weight * col_coef.values, col_coef, col_sig
```

SYNTAX USED TO OBTAIN THE TARGET EIGENVECTOR CENTRALITY

```
graph = nx.from_pandas_edgelist(df_main, source="source", target="destiny", edge_attr=["weight"])
targeted_eigenvector = nx.eigenvector_centrality(graph, weight=weight)
```
